# Timed Negotiations

**DOI:** 10.1007/978-3-030-45231-5_3

**Published:** 2020-04-17

**Authors:** S. Akshay, Blaise Genest, Loïc Hélouët, Sharvik Mital

**Affiliations:** 8 ENS/CNRS, Université Paris-Saclay, Cachan, France; 9 University of Duisburg-Essen, Duisburg, Germany; 10 IIT Bombay, Mumbai, India; 11 Univ Rennes, CNRS, IRISA, Rennes, France; 12 Univ Rennes, Inria, Rennes, France

## Abstract

Negotiations were introduced in [6] as a model for concurrent systems with multiparty decisions. What is very appealing with negotiations is that it is one of the very few non-trivial concurrent models where several interesting problems, such as soundness, i.e. absence of deadlocks, can be solved in PTIME [3]. In this paper, we introduce the model of timed negotiations and consider the problem of computing the minimum and the maximum execution times of a negotiation. The latter can be solved using the algorithm of [10] computing costs in negotiations, but surprisingly minimum execution time cannot.

This paper proposes new algorithms to compute both minimum and maximum execution time, that work in much more general classes of negotiations than [10], that only considered sound and deterministic negotiations. Further, we uncover the precise complexities of these questions, ranging from PTIME to $\varDelta _2^P$-complete. In particular, we show that computing the minimum execution time is more complex than computing the maximum execution time in most classes of negotiations we consider.

## 1 Introduction

Distributed systems are notoriously difficult to analyze, mainly due to the explosion of the number of configurations that have to be considered to answer even simple questions. A challenging task is then to propose models on which analysis can be performed with tractable complexities, preferably within polynomial time. Free choice Petri nets are a classical model of distributed systems that allow for efficient verification, in particular when the nets are 1-safe [4,5].

Recently, [6] introduced a new model called *negotiations* for workflows and business processes. A negotiation describes how processes interact in a distributed system: a subset of processes in a node of the system take a synchronous decisions among several *outcomes*. The effect of this outcome sends contributing processes to a new set of nodes. The execution of a negotiation ends when processes reach a *final configuration*. Negotiations can be deterministic (once an outcome is fixed, each process knows its unique successor node) or not.

Negotiations are an interesting model since several properties can be decided with a reasonable complexity. The question of *soundness*, i.e., deadlock-freedom:

* Supported by DST/CEFIPRA/INRIA Associated team EQuaVE and DST/SERB Matrices grant MTR/2018/000744.

whether from every reachable configuration one can reach a final configuration, is PSPACE-complete. However, for deterministic negotiations, it can be decided in PTIME [7]. The decision procedure uses reduction rules. Reduction techniques were originally proposed for Petri nets [2, 8, 11, 16]. The main idea is to define transformations rules that produce a model of smaller size w.r.t. the original model, while preserving the property under analysis. In the context of negotiations, [7, 3] proposed a sound and complete set of soundness-preserving reduction rules and algorithms to apply these rules efficiently. The question of soundness for deterministic negotiations was revisited in [9] and showed NLOGSPACE-complete using anti patterns instead of reduction rules. Further, they show that the PTIME result holds even when relaxing determinism [9]. Negotiation games have also been considered to decide whether one particular process can force termination of a negotiation. While this question is EXPTIME-complete in general, for sound and deterministic negotiations, it becomes PTIME [12].

While it is natural to consider cost or time in negotiations (e.g. think of the Brexit negotiation where time is of the essence, and which we model as running example in this paper), the original model of negotiations proposed by [6] is only qualitative. Recently, [10] has proposed a framework to associate costs to the executions of negotiations, and adapt a static analysis technique based on reduction rules to compute end-to-end cost functions that are not sensitive to scheduling of concurrent nodes. For sound *and* deterministic negotiations, the end-to-end cost can be computed in $O(n.(C + n))$, where $n$ is the size of the negotiation and $C$ the time needed to compute the cost of an execution. Requiring soundness or determinism seems perfectly reasonable, but asking sound *and* deterministic negotiations is too restrictive: it prevents a process from waiting for decisions of other processes to know how to proceed.

In this paper, we revisit time in negotiations. We attach time intervals to outcomes of nodes. We want to compute maximal and minimal executions times, for negotiations that are not necessarily sound and deterministic. Since we are interested in minimal and maximal execution time, cycles in negotiations can be either bypassed or lead to infinite maximal time. Hence, we restrict this study to acyclic negotiations. Notice that time can be modeled as a cost, following [10], and the maximal execution time of a sound and deterministic negotiation can be computed in PTIME using the algorithm from [10]. Surprisingly however, we give an example (Example 3) for which the minimal execution time cannot be computed in PTIME by this algorithm.

The first contribution of the paper shows that reachability (whether at least one run of a negotiation terminates) is NP-complete, already for (untimed) deterministic acyclic negotiations. This implies that computing minimal or maximal execution time for deterministic (but unsound) acyclic negotiations cannot be done in PTIME (unless NP=PTIME). We characterize precisely the complexities of different decision variants (threshold, equality, etc.), with complexities ranging from (co-)NP-complete to $\Delta_2^P$.

We thus turn to negotiations that are sound but not necessarily deterministic. Our second contribution is a new algorithm, not based on reduction rules, to compute the maximal execution time in PTIME for sound negotiations. It is based on computing the maximal execution time of critical paths in the negotiations. However, we show that *minimal* execution time cannot be computed in PTIME for sound negotiations (unless NP=PTIME): deciding whether the minimal execution time is lower than $T$ is NP-complete, even for $T$ given in unary, using a reduction from a Bin packing problem. This shows that minimal execution time is harder to compute than maximal execution time.

Our third contribution consists in defining a class in which the minimal execution time can be computed in (pseudo) PTIME. To do so, we define the class of $k$-layered negotiations, for $k$ fixed, that is negotiations where nodes can be organized into layers of at most $k$ nodes at the same depth. These negotiations can be executed without remembering more than $k$ nodes at a time. In this case, we show that computing the maximal execution time is PTIME, even if the negotiation is neither deterministic nor sound. The algorithm, not based on reduction rules, uses the $k$-layer restriction in order to navigate in the negotiation while considering only a polynomial number of configurations. For minimal execution time, we provide a pseudo PTIME algorithm, that is PTIME if constants are given in unary. Finally, we show that the size of constants do matter: deciding whether the minimal execution time of a $k$-layered negotiation is less than $T$ is NP-complete, when $T$ is given in binary. We show this by reducing from a Knapsack problem, yet again emphasizing that the minimal execution time of a negotiation is harder to compute than its maximal execution time.

This paper is organized as follows. Section 2 introduces the key ingredients of negotiations, determinism and soundness, known results in the untimed setting, and provides our running example modeling the Brexit negotiation. Section 3 introduces time in negotiations, gives a semantics to this new model, and formalizes several decision problems on maximal and minimal durations of runs in timed negotiations. We recall the main results of the paper in Section 4. Then, Section 5 considers timed execution problems for deterministic negotiations, Section 6 for sound negotiations, and section 7 for layered negotiations. Proof details for the last three sections are given in an extended version of this paper [1].

## 2 Negotiations: Definitions and Brexit example

In this section, we recall the definition of negotiations, of some subclasses (acyclic and deterministic), as well as important problems (soundness and reachability).

**Definition 1 (Negotiation [6, 10]).** *A* negotiation *over a finite set of processes $P$ is a tuple $\mathcal{N} = (N, n_0, n_f, \mathcal{X})$, where:*

- *$N$ is a finite set of nodes. Each node is a pair $n = (P_n, R_n)$ where $P_n \subseteq P$ is a non empty set of processes participating in node $n$, and $R_n$ is a finite set of outcomes of node $n$ (also called results), with $R_{n_f} = \{r_f\}$. We denote by $R$ the union of all outcomes of nodes in $N$.*
- *$n_0$ is the first node of the negotiation and $n_f$ is the final node. Every process in $P$ participates in both $n_0$ and $n_f$.*

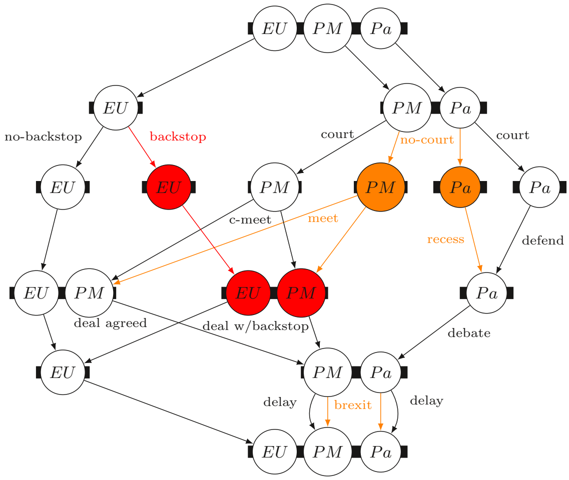

**Fig. 1.** A (sound but non-deterministic) negotiation modeling Brexit.

– *For all* $n \in N$, $\mathcal{X}_n : P_n \times R_n \to 2^N$ *is a map defining the transition relation from node* $n$, *with* $\mathcal{X}_n(p, r) = \emptyset$ *iff* $n = n_f, r = r_f$. *We denote* $\mathcal{X} : N \times P \times R \to 2^N$ *the partial map defined on* $\bigcup_{n \in N}(\{n\} \times P_n \times R_n)$, *with* $\mathcal{X}(n, p, a) = \mathcal{X}_n(p, a)$ *for all* $p, a$.

Intuitively, at a node $n = (P_n, R_n)$ in a negotiation, all processes of $P_n$ have to agree on a common outcome $r$ chosen from $R_n$. Once this outcome $r$ is chosen, every process $p \in P_n$ is ready to move to any node prescribed by $\mathcal{X}(n, p, r)$. A new node $m$ can only start when all processes of $P_m$ are ready to move to $m$.

*Example 1.* We illustrate negotiations by considering a simplified model of the Brexit negotiation, see Figure 1. There are 3 processes, $P = \{EU, PM, Pa\}$. At first $EU$ decides whether or not to enforce a backstop in any deal (outcome backstop) or not (outcome no-backstop). In the meantime, $PM$ decides to proroge $Pa$, and $Pa$ can choose or not to appeal to court (outcome court/no court). If it goes to court, then $PM$ and $Pa$ will take some time in court (c-meet, defend), before $PM$ can meet $EU$ to agree on a deal. Otherwise, $Pa$ goes to recess, and $PM$ can meet $EU$ directly. Once $EU$ and $PM$ agreed on a deal, $PM$ tries to convince $Pa$ to vote the deal. The final outcome is whether the deal is voted, or whether Brexit is delayed.

**Definition 2 (Deterministic negotiations).** *A process* $p \in P$ *is* deterministic *iff, for every* $n \in N$ *and every outcome* $r$ *of* $n$, $\mathcal{X}(n, p, r)$ *is a singleton. A negotiation is* deterministic *iff all its processes are deterministic. It is* weakly non-deterministic *[9] (called weakly deterministic in [3]) iff, for every node* $n$, *one of the processes in* $P_n$ *is deterministic. Last, it is* very weakly non-deterministic [9] *(called weakly deterministic in [6]) iff, for every* $n$, *every* $p \in P_n$ *and every outcome* $r$ *of* $n$, *there exists a deterministic process* $q$ *such that* $q \in P_{n'}$ *for every* $n' \in \mathcal{X}(n, p, r)$.

In deterministic negotiations, once an outcome is chosen, each process knows the next node it will be involved in. In (very-)weakly non-deterministic negotiations, the next node might depend upon the outcome chosen in other nodes by other processes. However, once the outcomes have been chosen for all current nodes, there is only one next node possible for each process. Observe that the class of deterministic negotiations is isomorphic to the class of free choice workflow nets [10]. In Example 1, the Brexit negotiation is non-deterministic, because process $PM$ is non-deterministic. Indeed, consider outcomes *c-meet*: it allows two nodes, according to whether the backstop is enforced or not, which is a decision taken by process $EU$.

**Semantics:** A *configuration* [3] of a negotiation is a mapping $M : P \to 2^N$. Intuitively, it tells for each process $p$ the set $M(p)$ of nodes $p$ is ready to engage in. The semantics of a negotiation is defined in terms of moves from a configuration to the next one. The *initial* $M_0$ and *final* $M_f$ configurations, are given by $M_0(p) = \{n_0\}$ and $M_f(p) = \emptyset$ respectively for every process $p \in P$. A configuration $M$ *enables* node $n$ if $n \in M(p)$ for every $p \in P_n$. When $n$ is enabled, a decision at node $n$ can occur, and the participants at this node choose an outcome $r \in R_n$. The occurrence of $(n, r)$ produces the configuration $M'$ given by $M'(p) = \mathcal{X}(n, p, r)$ for every $p \in P_n$ and $M'(p) = M(p)$ for remaining processes in $P \setminus P_n$. Moving from $M$ to $M'$ after choosing $(n, r)$ is called a *step*, denoted $M \xrightarrow{n,r} M'$. A *run* of $\mathcal{N}$ is a sequence $(n_1, r_1), (n_2, r_2)...(n_k, r_k)$ such that there is a sequence of configurations $M_0, M_1, \ldots, M_k$ and every $(n_i, r_i)$ is a step between $M_{i-1}$ and $M_i$. A run starting from the initial configuration and ending in the final configuration is called a *final run*. By definition, its last step is $(n_f, r_f)$.

An important class of negotiations in the context of timed negotiations is acyclic negotiations, where infinite sequence of steps is impossible:

**Definition 3 (Acyclic negotiations).** *The* graph *of a negotiation* $\mathcal{N}$ *is the labeled graph* $G_{\mathcal{N}} = (V, E)$ *where* $V = N$*, and* $E = \{((n, (p, r), n') \mid n' \in \mathcal{X}(n, p, r)\}$*, with pairs of the form* $(p, r)$ *being the labels. A negotiation is* acyclic *iff its graph is acyclic. We denote by* $Paths(G_{\mathcal{N}})$ *the set of paths in the graph of a negotiation. These paths are of form* $\pi = (n_0, (p_0, r_0), n_1) \ldots (n_{k-1}, (p_k, r_k), n_k)$.

The Brexit negotiation of Fig.1 is an example of acyclic negotiation. Despite their apparent simplicity, negotiations may express involved behaviors as shown with the Brexit example. Indeed two important questions in this setting are whether there is some way to reach a final node in the negotiation from (i) the initial node and (ii) any reachable node in the negotiation.

**Definition 4 (Soundness and Reachability).**

1. *A negotiation is* sound *iff every run from the initial configuration can be extended to a final run. The problem of soundness is to check if a given negotiation is sound.*
2. *The problem of reachability asks if a given negotiation has a final run.*

Notice that the Brexit negotiation of Fig.1 is sound (but not deterministic). It seems hard to preserve the important features of this negotiation while being both sound *and* deterministic. The problem of soundness has received considerable attention. We summarize the results about soudness in the next theorem:

**Theorem 1.** *Determining whether a negotiation is sound is PSPACE-Complete. For (very-)weakly non-deterministic negotiations, it is co-NP-complete [9]. For acyclic negotiations, it is in DP and co-NP-Hard [6]. Determining whether an acyclic weakly non-deterministic negotiation is sound is in PTIME [3, 9]. Finally, deciding soundness for deterministic negotiations is NLOGSPACE-complete [9].*

Checking reachability is NP-complete, even for deterministic acyclic negotiations (surprisingly, we did not find this result stated before in the literature):

**Proposition 1.** *Reachability is NP-complete for acyclic negotiations, even if the negotiation is deterministic.*

*Proof (sketch).* One can guess a run of size $\leq |\mathcal{N}|$ in polynomial time, and verify if it reaches $n_f$, which gives the inclusion in NP. The hardness part comes from a reduction from 3-CNF-SAT that can be found in the proof of Theorem 3. □

## *k*-Layered Acyclic Negotiations

We introduce a new class of negotiations which has good algorithmic properties, namely $k$-layered acyclic negotiations, for $k$ fixed. Roughly speaking, nodes of a $k$-layered acyclic negotiations can be arranged in layers, and these layers contain at most $k$ nodes. Before giving a formal definition, we need to define the depth of nodes in $\mathcal{N}$.

First, a *path* in a negotiation is a sequence of nodes $n_0 \dots n_\ell$ such that for all $i \in \{1, \dots, \ell-1\}$, there exists $p_i, r_i$ with $n_{i+1} \in \mathcal{X}(n_i, p_i, r_i)$. The *length* of a path $n_0, \dots, n_\ell$ is $\ell$. The *depth* $\mathrm{depth}(n)$ of a node $n$ is the maximal length of a path from $n_0$ to $n$ (recall that $\mathcal{N}$ is acyclic, so this number is always finite).

**Definition 5.** *An acyclic negotiation is* layered *if for all node $n$, every path reaching $n$ has length depth($n$). An acyclic negotiation is $k$-*layered *if it is layered, and for all $\ell \in \mathbb{N}$, there are at most $k$ nodes at depth $\ell$.*

The Brexit example of Fig. 1 is 6-layered. Notice that a layered negotiation is necessarily $k$-layered for some $k \leq |\mathcal{N}| - 2$. Note also that we can always transform an acyclic negotiation $\mathcal{N}$ into a layered acyclic negotiation $\mathcal{N}'$, by adding dummy nodes: for every node $m \in \mathcal{X}(n, p, r)$ with $\mathrm{depth}(m) > \mathrm{depth}(n)+1$, we can add several nodes $n_1, \dots n_\ell$ with $\ell = \mathrm{depth}(m) - (\mathrm{depth}(n) + 1)$, and processes $P_{n_i} = \{p\}$. We compute a new relation $\mathcal{X}'$ such that $\mathcal{X}'(n, p, r) = \{n_1\}$, $\mathcal{X}(n_\ell, p, r) = \{m\}$ and for every $i \in 1..\ell - 1$, $\mathcal{X}(n_i, p, r) = n_{i+1}$. This transformation is polynomial: the resulting negotiation is of size up to $|\mathcal{N}| \times |\mathcal{X}| \times |P|$. The proof of the following Theorem can be found in [1].

**Theorem 2.** *Let $k \in \mathbb{N}^+$. Checking reachability or soundness for a $k$-layered acyclic negotiation $\mathcal{N}$ can be done in PTIME.*

## 3 Timed Negotiations

In many negotiations, time is an important feature to take into account. For instance, in the Brexit example, with an initial node starting at the begining of September 2019, there are 9 weeks to pass a deal till the $31^{st}$ October deadline.

We extend negotiations by introducing timing constraints on outcomes of nodes, inspired by timed Petri nets [14] and by the notion of negotiations with costs [10]. We use time intervals to specify lower and upper bounds for the duration of negotiations. More precisely, we attach time intervals to pairs $(n, r)$ where $n$ is a node and $r$ an outcome. In the rest of the paper, we denote by $\mathcal{I}$ the set of intervals with endpoints that are non-negative integers or $\infty$. For convenience we only use closed intervals in this paper (except for $\infty$), but the results we show can also be extended to open intervals with some notational overhead. Intuitively, outcome $r$ can be taken at a node $n$ with associated time interval $[a, b]$ only after $a$ time units have elapsed from the time all processes contributing to $n$ are ready to engage in $n$, and at most $b$ time units later.

**Definition 6.** *A* timed negotiation *is a pair* $(\mathcal{N}, \gamma)$ *where* $\mathcal{N}$ *is a negotiation, and* $\gamma : N \times R \to \mathcal{I}$ *associates an interval to each pair* $(n, r)$ *of node and outcome such that* $r \in R_n$*. For a given node* $n$ *and outcome* $r$*, we denote by* $\gamma^-(n, r)$ *(resp.* $\gamma^+(n, r)$*) the lower bound (resp. the upper bound) of* $\gamma(n, r)$*.*

*Example 2.* In the Brexit example, we define the following timed constraints $\gamma$. We only specify the outcome names, as the timing only depends upon them. Backstop and no-backstop both take between 1 and 2 weeks: $\gamma(\text{backstop}) = \gamma(\text{no-backstop}) = [1, 2]$. In case of no-court, recess takes 5 weeks $\gamma(\text{recess}) = [5, 5]$, and PM can meet EU immediatly $\gamma(\text{meet}) = [0, 0]$. In case of court action, PM needs to spend 2 weeks in court $\gamma(\text{c-meet}) = [2, 2]$, and depending on the court delay and decision, Pa needs between 3 (court overules recess) to 5 (court confirms recess) weeks, $\gamma(\text{defend}) = [3, 5]$. Agreeing on a deal can take anywhere from 2 weeks to 2 years (104 weeks): $\gamma(\text{deal agreed}) = [2, 104]$—some would say infinite time is even possible! It needs more time with the backstop, $\gamma(\text{deal w/backstop}) = [5, 104]$. All other outcomes are assumed to be immediate, i.e., associated with $[0, 0]$.

**Semantics:** A *timed valuation* is a map $\mu : P \to \mathbb{R}^{\geq 0}$ that associates a non-negative real value to every process. A *timed configuration* is a pair $(M, \mu)$ where $M$ is a configuration and $\mu$ a timed valuation. There is a *timed step* from $(M, \mu)$ to $(M', \mu')$, denoted $(M, \mu) \xrightarrow{(n,r)} (M', \mu')$, if (i) $M \xrightarrow{(n,r)} M'$, (ii) $p \notin P_n$ implies $\mu'(p) = \mu(p)$ (iii) $\exists d \in \gamma(n, r)$ such that $\forall p \in P_n$, we have $\mu'(p) = \max_{p' \in P_n} \mu(p') + d$ ($d$ is the duration of node $n$).

Intuitively a timed step $(M, \mu) \xrightarrow{(n,r)} (M', \mu')$ depicts a decision taken at node $n$, and how long each process of $P_n$ waited in that node before taking decision $(n, r)$. The last process engaged in $n$ must wait for a duration contained in $\gamma(n, r)$. However, other processes may spend a time greater than $\gamma^+(n, r)$.

A *timed run* is a sequence of steps $\rho = (M_0, \mu_0) \xrightarrow{e_1} (M_1, \mu_1) \dots (M_k, \mu_k)$ where $M_0$ is the initial configuration, $\mu_0(p) = 0$ for every $p \in P$, and each $(M_i, \mu_i) \xrightarrow{e_i} (M_{i+1}, \mu_{i+1})$ is a timed step. It is *final* if $M_k = M_f$. Its *execution time* $\delta(\rho)$ is defined as $\delta(\rho) = \max_{p \in P} \mu_k(p)$.

Notice that we only attached timing to processes, not to individual steps. With our definition of runs, timing on steps may not be monotonous (i.e., non-decreasing) along the run, while timing on processes is. Viewed by the lens of concurrent systems, the timing is monotonous on the partial orders of the system rather than the linearization. It is not hard to restrict paths, if necessary, to have a monotonous timing on steps as well. In this paper, we are only interested in execution time, which does not depend on the linearization considered.

Given a timed negotiation $\mathcal{N}$, we can now define the minimum and maximum execution time, which correspond to optimistic or pessimistic views:

**Definition 7.** *Let $\mathcal{N}$ be a timed negotiation. Its* minimum execution time*, denoted mintime($\mathcal{N}$) is the minimal $\delta(\rho)$ over all final timed run $\rho$ of $\mathcal{N}$. We define the* maximal execution time *maxtime($\mathcal{N}$) of $\mathcal{N}$ similarly.*

Given $T \in \mathbb{N}$, the main problems we consider in this paper are the following:

- The mintime problem, i.e., do we have $mintime(\mathcal{N}) \leq T$**?**.
  In other words, does there exist a final timed run $\rho$ with $\delta(\rho) \leq T$?
- The maxtime problem, i.e., do we have $maxtime(\mathcal{N}) \leq T$**?**.
  In other words, does $\delta(\rho) \leq T$ for every final timed run $\rho$?

These questions have a practical interest : in the Brexit example, the question "is there a way to have a vote on a deal within 9 weeks ?" is indeed a minimum execution time problem. We also address the equality variant of these decision problems, i.e., $mintime(\mathcal{N}) = T$ **:** is there a final run of $\mathcal{N}$ that terminates in exactly $T$ time units and no other final run takes less than $T$ time units? Similarly for $maxtime(\mathcal{N}) = T$.

*Example 3.* We use Fig. 1 to show that it is not easy to compute the minimal execution time, and in particular one cannot use the algorithm from [10] to compute it. Consider the node $n$ with $P_n = \{PM, Pa\}$ and $R_n = \{\text{court}, \text{no\_court}\}$. If the outcome is court, then $PM$ needs 2 weeks before (s)he can talk to $EU$ and $Pa$ needs at least 3 weeks before he can debate. However, if the outcome is no_court, then $PM$ need not wait before (s)he can talk to $EU$, but $Pa$ wastes 5 weeks in recess. This means that one needs to remember different alternatives which could be faster in the end, depending on the future. On the other hand, the algorithm from [10] attaches one minimal time to process $Pa$, and one minimal time to process $PM$. No matter the choices (0 or 2 for $PM$ and 3 or 5 for $Pa$), there will be futures in which the chosen number will over or underapproximate the real minimal execution time (this choice is not explicit in [10])[4].

[4] the authors of [10] acknowledged the issue with their algorithm for mintime.

For maximum execution time, it is not an issue to attach to each node a unique maximal execution time. The reason for the asymmetry between minimal and maximal execution times of a negotiation is that the execution time of a path is $\max_{p \in P} \mu_k(p)$, for $\mu_k$ the last timed valuation, which breaks the symmetry between min and max.

## 4 High level view of the main results

In this section, we give a high-level description of our main results. Formal statements can be found in the sections where they are proved. We gather in Fig. 2 the precise complexities for the minimal and the maximal execution time problems for 3 classes of negotiations that we describe in the following. Since we are interested in minimum and maximum execution time, cycles in negotiations can be either bypassed or lead to infinite maximal time. Hence, while we define timed negotiations in general, we always restrict to acyclic negotiations (such as Brexit) while stating and proving results.

In [10], a PTIME algorithm is given to compute different costs for negotiations that are both sound *and* deterministic. One limitation of this result is that it cannot compute the minimum execution time, as explained in Example 3. A second limitation is that the class of sound and deterministic negotiations is quite restrictive: it cannot model situations where the next node a process participates in depends on the outcome from another process, as in the Brexit example. We thus consider classes where one of these restrictions is dropped.

We first consider (Section 5) negotiations that are deterministic, but without the soundness restriction. We show that for this class, no timed problem we consider can be solved in PTIME (unless NP=PTIME). Further, we show that the equality problems ($maxtime/mintime(\mathcal{N}) = T$), are complete for the complexity class DP, i.e., at the second level of the Boolean Hierarchy [15].

We then consider (Section 6) the class of negotiations that are sound, but not necessarily deterministic. We show that maximum execution time can be solved in PTIME, and propose a new algorithm. However, the minimum execution time cannot be computed in PTIME (unless NP=PTIME). Again for the mintime equality problem we have a matching DP-completeness result.

| | Deterministic | Sound | $k$-layered |
|---|---|---|---|
| Max $\leq T$<br>Max $= T$ | co-NP-complete (Thm. 3)<br>DP-complete (Prop. 2) | PTIME (Prop. 3) | PTIME (Thm. 6) |
| Min $\leq T$ | NP-complete (Thm. 3) | NP-complete$^\star$ (Thm. 5) | pseudo-PTIME (Thm. 8)<br>NP-complete$^{\star\star}$ (Thm. 7) |
| Min $= T$ | DP-complete (Prop. 2) | DP-complete$^\star$ (Prop. 4) | pseudo-PTIME (Thm. 8) |

**Fig. 2.** Results for acyclic timed negotiations. *DP* refers to the complexity class, Difference Polynomial time [15], the second level of the Boolean Hierarchy.
$^\star$ hardness holds even for very weakly non-deterministic negotiations, and $T$ in unary.
$^{\star\star}$ hardness holds even for sound and very weakly non-deterministic negotiations.

Finally, in order to obtain a polytime algorithm to compute the minimum execution time, we consider the class of $k$-layered negotiations (see Section 7): Given $k \in \mathbb{N}$, we can show that $maxtime(\mathcal{N})$ can be computed in PTIME for $k$-layered negotiations. We also show that while the $mintime(\mathcal{N}) \leq T$? problem is weakly NP-complete for $k$-layered negotiations, we can compute $mintime(\mathcal{N})$ in pseudo-PTIME, i.e. in PTIME if constants are given in unary.

## 5 Deterministic Negotiations

We start by considering the class of deterministic acyclic negotiations. We show that both maximal and minimal execution times cannot be computed in PTIME (unless NP=PTIME), as the threshold problems are (co-)NP-complete.

**Theorem 3.** *The $mintime(\mathcal{N}) \leq T$ decision problem is NP complete, and the $maxtime(\mathcal{N}) \leq T$ decision problem is co-NP-complete for acyclic deterministic timed negotiations.*

*Proof.* For $mintime(\mathcal{N}) \leq T$, containment in NP is easy: we just need to guess a run $\rho$ (of polynomial size as $\mathcal{N}$ is acyclic), consider the associated timed run $\rho^-$ where all decisions are taken at their earliest possible dates, and check whether $\delta(\rho^-) \leq T$, which can be done in time $O(|\mathcal{N}|+\log T)$.

For the hardness, we give the proof in two steps. First, we start with a proof of Proposition 1 that reachability problem is NP-hard using reduction of 3-CNF SAT, i.e., given a formula $\phi$, we build a deterministic negotiation $\mathcal{N}_\phi$ s.t. $\phi$ is satisfiable iff $\mathcal{N}_\phi$ has a final run. In a second step, we introduce timings on this negotiation and show that $mintime(\mathcal{N}_\phi) \leq T$ iff $\phi$ is satisfiable.

*Step 1: Reducing 3-CNF-SAT to Reachability problem.*

Given a Boolean formula $\phi$ with variables $v_i, 1 \leq i \leq n$ and clauses $c_j, 1 \leq j \leq m$, for each variable $v_i$ we define the sets of clauses $S_{i,\mathtt{t}} = \{c_j \mid v_i \text{ is present in } c_j\}$ and $S_{i,\mathtt{f}} = \{c_j \mid \neg v_i \text{ is present in } c_j\}$. Clauses in $S_{i,\mathtt{t}}$ and $S_{i,\mathtt{f}}$ are naturally ordered: $c_i < c_j$ iff $i < j$. We denote these elements $S_{i,\mathtt{t}}(1) < S_{i,\mathtt{t}}(2) < \ldots$. Similarly for set $S_{i,\mathtt{f}}$.

Now, we construct a negotiation $\mathcal{N}_\phi$ (as depicted in Figure 3) with a process $V_i$ for each variable $v_i$ and a process $C_j$ for each clause $c_j$:

- Initial node $n_0$ has a single outcome $r$ taking each process $C_j$ to node $Lone_{c_j}$, and each process $V_i$ to node $Lone_{v_i}$.
- $Lone_{c_j}$ has three outcomes: if literal $v_i \in c_j$, then $t_i$ is an outcome, taking $C_j$ to $Pair_{c_j,v_i}$, and if literal $\neg v_i \in c_j$, then $f_i$ is an outcome, taking $C_j$ to $Pair_{c_j,\neg v_i}$.
- The outcomes of $Lone_{v_i}$ are `true` and `false`. Outcome `true` brings $V_i$ to node $Tlone_{v_i,1}$ and outcome `false` brings $V_i$ to node $Flone_{v_i,1}$.
- We have a node $Tlone_{v_i,j}$ for each $j \leq |S_{i,\mathtt{t}}|$ and $Flone_{v_i,j}$ for each $j \leq |S_{i,\mathtt{f}}|$, with $V_i$ as only process. Let $c_r = S_{i,\mathtt{t}}(j)$. Node $Tlone_{v_i,j}$ has two outcomes $vton$ bringing $V_i$ to $Tlone_{v_i,j+1}$ (or $n_f$ if $j = |S_{i,\mathtt{t}}|$), and $vtoc_{i,r}$ bringing $V_i$ to $Pair_{c_r,v_i}$. The two outcomes from $Flone_{v_i,j}$ are similar.

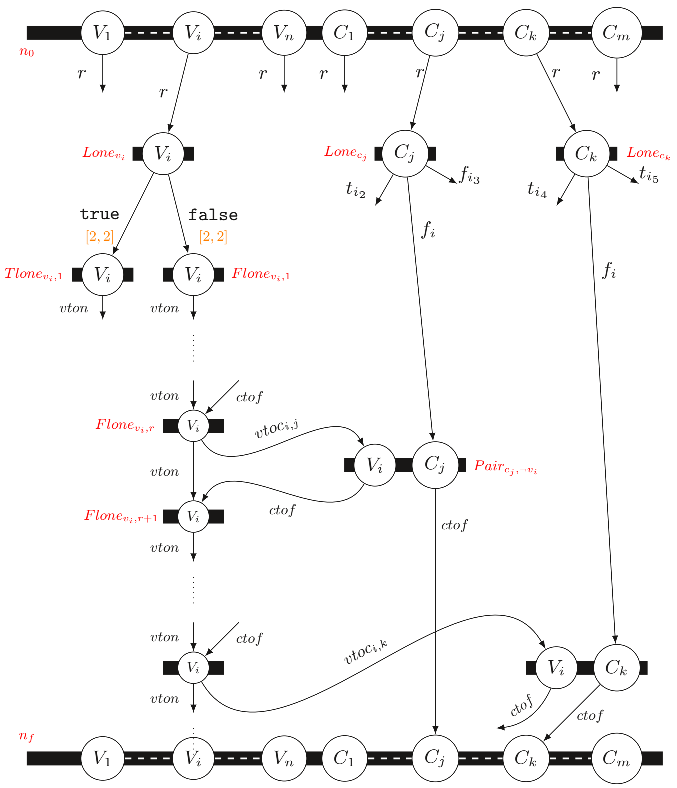

**Fig. 3.** A part of $\mathcal{N}_\phi$ where clause $c_j$ is $(i_2 \vee \neg i \vee \neg i_3)$ and clause $c_k$ is $(i_4 \vee \neg i \vee i_5)$. Timing is $[0, 0]$ whereever not mentioned

– Node $Pair_{c_r,v_i}$ has $V_i$ and $C_r$ as its processes and one outcome $ctof$ which takes process $C_r$ to final node $n_f$ and process $V_i$ to $Tlone_{v_i,j+1}$ (with $c_r = S_{i,\mathtt{t}}(j)$), or to $n_f$ if $j = |S_{i,\mathtt{t}}|$. Node $Pair_{c_r,\neg v_i}$ is defined in the same way from $Flone_{v_i,j}$.

With this we claim that $\mathcal{N}_\phi$ has a final run iff $\phi$ is satisfiable which completes the first step of the proof. We give a formal proof of this claim in Appendix A of [1]. Observe that the negotiation $\mathcal{N}_\phi$ constructed is deterministic and acyclic (but it is not sound).

*Step 2*: Before we introduce timing on $\mathcal{N}_\phi$, we introduce a new outcome $r'$ at $n_0$ which takes all processes to $n_f$. Now, the timing function $\gamma$ associated with $\mathcal{N}_\phi$ is: $\gamma(n_0, r) = [2, 2]$ and $\gamma(n_0, r') = [3, 3]$ and $\gamma(n, r) = [0, 0]$, for all node $n \neq n_0$ and all $r \in R_n$. Then, $mintime(\mathcal{N}_\phi) \leq 2$ iff $\phi$ has a satisfiable assignment: if $mintime(\mathcal{N}_\phi) \leq 2$, there is a run with decision $r$ taken at $n_0$ which is final. But existence of any such final run implies satisfiability of $\phi$. For reverse implication, if $\phi$ is satisfiable, then the corresponding run for satisfying assignment takes 2 time units, which means that $mintime(\mathcal{N}_\phi) \leq 2$.

Similarly, we can prove that the MaxTime problem is co-NP complete by changing $\gamma(n_0, r^{'}) = [1,1]$ and asking if $maxtime(\mathcal{N}_\phi) > 1$ for the new $\mathcal{N}_\phi$. The answer will be yes iff $\phi$ is satisfiable. □

We now consider the related problem of checking if $mintime(\mathcal{N}) = T$ (or if $maxtime(\mathcal{N}) = T$). These problems are harder than their threshold variant under usual complexity assumptions: they are DP-complete (Difference Polynomial time class, i.e., second level of the Boolean Hierarchy, defined as intersection of a problem in NP and one in co-NP [15]).

**Proposition 2.** *The $mintime(\mathcal{N}) = T$ and $maxtime(\mathcal{N}) = T$ decision problems are DP-complete for acyclic deterministic negotiations.*

*Proof.* We only give the proof for *mintime* (the proof for *maxtime* is given in Appendix A of [1]). Indeed, it is easy to see that this problem is in DP, as it can be written as $mintime(\mathcal{N}) \leq T$ which is in NP and $\neg(mintime(\mathcal{N}) \leq T-1))$, which is in co-NP. To show hardness, we use the negotiation constructed in the above proof as a gadget, and show a reduction from the SAT-UNSAT problem (a standard DP-complete problem).

The SAT-UNSAT Problem asks given two Boolean expressions $\phi$ and $\phi^{'}$, both in CNF forms with three literals per clause, is it true that $\phi$ is satisfiable and $\phi^{'}$ is unsatisfiable? SAT-UNSAT is known to be DP-complete [15]. We reduce this problem to $mintime(\mathcal{N}) = T$.

Given $\phi$, $\phi^{'}$, we first make the corresponding negotiations $\mathcal{N}_\phi$ and $\mathcal{N}_{\phi'}$ as in the previous proof. Let $n_0$ and $n_f$ be the initial and final nodes of $\mathcal{N}_\phi$ and $n_0^{'}$ and $n_f^{'}$ be the initial and final nodes of $\mathcal{N}_{\phi'}$. (Similarly, for other nodes we write $'$ above the nodes to signify they belong to $\mathcal{N}_{\phi'}$.)

In the negotiation $\mathcal{N}_{\phi'}$, we introduce a new node $n_{all}$, in which all the processes participate (see Figure 4). The node $n_{all}$ has a single outcome $r'_{all}$ which sends all the processes to $n_f$. Also, for node $n_0^{'}$, apart from the outcome $r$ which sends all processes to different nodes, there is another outcome $r_{all}$ which sends all the processes to $n_{all}$. Now we merge the nodes $n_f$ and $n_0^{'}$ and call the merged node $n_{sep}$. Also nodes $n_0$ and $n'_f$ now have all the processes of $\mathcal{N}_\phi$ and $\mathcal{N}_{\phi'}$ participating in them. This merged process gives us a new negotiation $\mathcal{N}_{\phi,\phi'}$ in which the structure above $n_{sep}$ is same as $\mathcal{N}_\phi$ while below it is same as $\mathcal{N}_{\phi'}$. Node $n_{sep}$ now has all the processes of $\mathcal{N}_\phi$ and $\mathcal{N}_{\phi'}$ participating in it. The outcomes of $n_{sep}$ will be same as that of $n'_0$ $(r_{all}, r)$. For both the outcomes of $n_{sep}$ the processes corresponding to $\mathcal{N}_\phi$ directly go to $n_f$ of the $\mathcal{N}_{\phi,\phi'}$. Similarly $n_0$ of $\mathcal{N}_{\phi,\phi'}$ which is same $n_0$ of $\mathcal{N}_\phi$, sends processes corresponding to $\mathcal{N}_{\phi'}$ directly to $n_{sep}$ for all its outcomes. We now define timing function $\gamma$ for $\mathcal{N}_{\phi,\phi'}$ which is as follows: $\gamma(Lone^{'}_{v_i}, r) = [1,1]$ for all $v_i \in \phi^{'}$ and $r \in \{\texttt{true, false}\}$, $\gamma(n_{all}, r'_{all}) = [2,2]$ and $\gamma(n,r) = [0,0]$ for all other outcomes of nodes. With this construction, one can conclude that $mintime(\mathcal{N}_{\phi,\phi'}) = 2$ iff $\phi$ is satisfiable and $\phi^{'}$ is unsatisfiable (see [1] for details). This completes the reduction and hence proves DP-hardness. □

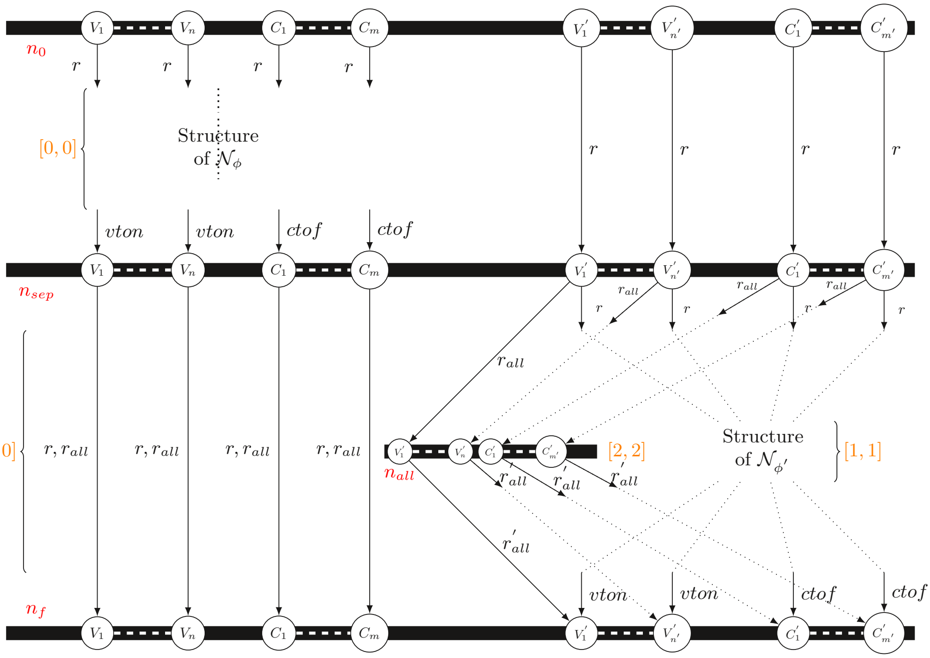

**Fig. 4.** Structure of $\mathcal{N}_{\phi,\phi'}$

Finally, we consider a related problem of computing the min and max time. To consider the decision variant, we rephrase this problem as checking whether an arbitrary bit of the minimum execution time is 1. Perhaps surprisingly, we obtain that this problem goes even beyond DP, the second level of the Boolean Hierarchy and is in fact hard for $\Delta_2^P$ (second level of the *polynomial* hierarchy), which contains the entire Boolean Hierarchy. Formally,

**Theorem 4.** *Given an acyclic deterministic timed negotiation and a positive integer* $k$*,computing the* $k^{th}$ *bit of the maximum/minimum execution time is* $\Delta_2^P$*-complete.*

Finally, we remark that if we were interested in the optimization variant and not the decision variant of the problem, the above proof can be adapted to show that these variants are OptP-complete (as defined in [13]). But as optimization is not the focus of this paper, we avoid formal details of this proof.

## 6 Sound Negotiations

Sound negotiations are negotiations in which every run can be extended to a final run, as in Fig. 1. In this section, we show that $maxtime(\mathcal{N})$ can be computed in PTIME for sound negotiations, hence giving PTIME complexities for the $maxtime(\mathcal{N}) \leq T$? and $maxtime(\mathcal{N}) = T$? questions. However, we show that $mintime(\mathcal{N}) \leq T$ is NP-complete for sound negotiations, and that $mintime(\mathcal{N}) = T$ is DP-complete, even if $T$ is given in unary.

Consider the graph $G_{\mathcal{N}}$ of a negotiation $\mathcal{N}$. Let $\pi = (n_0, (p_0, r_0), n_1) \cdots (n_k, (p_k, r_k), n_{k+1})$ be a path of $G_{\mathcal{N}}$. We define the *maximal execution time* of a path $\pi$ as the value $\delta^+(\pi) = \sum_{i \in 0..k} \gamma^+(n_i, r_i)$. We say that a path $\pi = (n_0, (p_0, r_0), n_1) \cdots (n_\ell, (p_\ell, r_\ell), n_{\ell+1})$ is a path of some run $\rho = (M_1, \mu_1) \xrightarrow{(n_1, r_1')} \cdots (M_k, \mu_k)$ if $r_0, \ldots, r_\ell$ is a subword of $r_1', \ldots, r_k'$.

**Lemma 1.** *Let $\mathcal{N}$ be an acyclic and sound timed negotiation. Then $maxtime(\mathcal{N}) = \max_{\pi \in Paths(G_{\mathcal{N}})} \delta^+(\pi) + \gamma^+(n_f, r_f)$.*

*Proof.* Let us first prove that $maxtime(\mathcal{N}) \geq \max_{\pi \in Paths(G_{\mathcal{N}})} \delta^+(\pi) + \gamma^+(n_f, r_f)$. Consider any path $\pi$ of $G_{\mathcal{N}}$, ending in some node $n$. First, as $\mathcal{N}$ is sound, we can compute a run $\rho_\pi$ such that $\pi$ is a path of $\rho_\pi$, and $\rho_\pi$ ends in a configuration in which $n$ is enabled. We associate with $\rho_\pi$ the timed run $\rho_\pi^+$ which associates to every node the latest possible execution date. We have easily $\delta(\rho_\pi^+) \geq \delta^+(\pi)$, and then we obtain $\max_{\pi \in Paths(G_{\mathcal{N}})} \delta(\rho_\pi^+) \geq \max_{\pi \in Paths(G_{\mathcal{N}})} \delta^+(\pi)$. As $maxtime(\mathcal{N})$ is the maximal duration over all runs, it is hence necessarily greater than $\max_{\pi \in Paths(G_{\mathcal{N}})} \delta(\rho_\pi^+) + \gamma^+(n_f, r_f)$.

We now prove that $maxtime(\mathcal{N}) \leq \max_{\pi \in Paths(G_{\mathcal{N}})} \delta^+(\pi) + \gamma^+(n_f, r_f)$. Take any timed run $\rho = (M_1, \mu_1) \xrightarrow{(n_1, r_1)} \cdots (M_k, \mu_k)$ of $\mathcal{N}$ with a unique maximal node $n_k$. We show that there exists a path $\pi$ of $\rho$ such that $\delta(\rho) \leq \delta^+(\pi)$ by induction on the length $k$ of $\rho$. The initialization is trivial for $k = 1$. Let $k \in \mathbb{N}$. Because $n_k$ is the unique maximal node of $\rho$, we have $\delta^+(\rho) = \max_{p \in P_{n_k}} \mu_{k-1}(p) + \gamma^+(n_k, r_k)$. We choose one $p_{k-1}$ maximizing $\mu_{k-1}(p)$. Let $\ell < k$ be the maximal index of a decision involving process $p_{k-1}$ (i.e. $p_{k-1} \in P_{n_\ell}$). Now, consider the timed run $\rho'$ subword of $\rho$, but with $n_\ell$ as unique maximal node (that is, it is $\rho$ where nodes $n_i, i > \ell$ has been removed, but also where some nodes $n_i, i < \ell$ have been removed if they are not causally before $n_\ell$ (in particular, $P_{n_i} \cap P_{n_\ell} = \emptyset$).)

By definition, we have that $\delta^+(\rho) = \delta^+(\rho') + \gamma^+(n_\ell, r_\ell) + \gamma^+(n_k, r_k)$. We apply the induction hypothesis on $\rho'$, and obtain a path $\pi'$ of $\rho'$ ending in $n_\ell$ such that $\delta^+(\rho') + \gamma^+(n_\ell, r_\ell) \leq \delta^+(\pi')$. It suffices to consider path $\pi = \pi'.(n_\ell, (p_{k-1}, r_\ell), n_k)$ to prove the inductive step $\delta^+(\rho) \leq \delta^+(\pi) + \gamma^+(n_k, r_k)$.

Thus $maxtime(\mathcal{N}) = \max \delta^+(\rho) \leq \max_{\pi \in Paths(G_{\mathcal{N}})} \delta^+(\pi) + \gamma^+(n_f, r_f)$. $\square$

Lemma 1 gives a way to evaluate the maximal execution time. This amounts to finding a path of maximal weight in an acyclic graph, which is a standard PTIME problem that can be solved using standard max-cost calculation.

**Proposition 3.** *Computing the maximal execution time for an acyclic sound negotiation $\mathcal{N} = (N, n_0, n_f, \mathcal{X})$ can be done in time $O(|N| + |\mathcal{X}|)$.*

A direct consequence is that $maxtime(\mathcal{N}) \leq T$ and $maxtime(\mathcal{N}) = T$ problems can be solved in polynomial time when $\mathcal{N}$ is sound. Notice that if $\mathcal{N}$ is deterministic but not sound, then Lemma 1 does not hold: we only have an inequality.

We now turn to *mintime*($\mathcal{N}$). We show that it is strictly harder to compute for sound negotiations than *maxtime*($\mathcal{N}$).

**Theorem 5.** *$mintime(\mathcal{N}) \leq T$ is NP-complete in the strong sense for sound acyclic negotiations, even if $\mathcal{N}$ is very weakly non-deterministic.*

*Proof (sketch).* First, we can decide $mintime(\mathcal{N}) \leq T$ in NP. Indeed, one can guess a final (untimed) run $\rho$ of size $\leq |N|$, consider $\rho^-$ the timed run corresponding to $\rho$ where all outcomes are taken at the earliest possible dates, and compute in linear time $\delta(\rho^-)$, and check that $\delta(\rho^-) \leq T$.

The hardness part is obtained by reduction from the **Bin Packing** problem. The reduction is similar to Knapsack, that we will present in Thm. 7. The difference is that we use $\ell$ bins in parallel, rather than 2 processes, one for the weight and one for the value. The hardness is thus strong, but the negotiation is not $k$-layered for a bounded $k$ (it is $2\ell + 1$ bounded, with $\ell$ depending on the input). A detailed proof is given in Appendix B of [1]. □

We show that $mintime(\mathcal{N}) = T$ is harder to decide than $mintime(\mathcal{N}) \leq T$, with a proof similar to Prop. 2.

**Proposition 4.** *The $mintime(\mathcal{N}) = T$? decision problem is DP-complete for sound acyclic negotiations, even if it is very weakly non-deterministic.*

An open question is whether the minimal execution time can be computed in PTIME if the negotiation is both sound and deterministic. The reduction from Bin Packing does not work with deterministic (and sound) negotiations.

## 7 *k*-Layered Negotiations

In this section, we consider $k$-layeredness, a syntactic property that can be efficiently verified (see Section 2).

### 7.1 Algorithmic properties

Let $k$ be a fixed integer. We first show that the maximum execution time can be computed in PTIME for $k$-layered negotiations. Let $N_i$ be the set of nodes at layer $i$. We define for every layer $i$ the set $S_i$ of subsets of nodes $X \subseteq N_i$ which can be jointly enabled and such that for every process $p$, there is exactly one node $n(X, p)$ in $X$ with $p \in n(X, p)$. An element $X$ in $S_i$ is a subset of nodes that can be selected by solving all non-determnism with an appropriate choice of outcomes. Formally, we define $S_i$ inductively. We start with $S_0 = \{n_0\}$. We then define $S_{i+1}$ from the contents of layer $S_i$: we have $Y \in S_{i+1}$ iff $\bigcup_{n \in Y} P_n = P$ and there exist $X \in S_i$ and an outcome $r_m \in R_m$ for every $m \in X$, such that $n \in \mathcal{X}(n(X,p), p, r_m)$ for each $n \in Y$ and $p \in P_n$.

**Theorem 6.** *Let $k \in \mathbb{N}^+$. Computing the maximum execution time for a $k$-layered acyclic negotiation $\mathcal{N}$ can be done in PTIME. More precisely, the worst-case time complexity is $O(|P| \cdot |\mathcal{N}|^{k+1})$.*

*Proof (Sketch).* The first step is to compute $S_i$ layer by layer, by following its inductive definition. The set $S_i$ is of size at most $2^k$, as $|N_i| < k$ by definition of $k$-layeredness. Knowing $S_i$, it is easy to build $S_{i+1}$ by induction. This takes time in $O(|P||\mathcal{N}|^{k+1})$ : We need to consider all $k$-uples of outcomes for each layer. There can be $|\mathcal{N}|^k$ such tuples. We need to do that for all processes ($|P|$), and for all layers (at most $|\mathcal{N}|$).

We then keep for each subset $X \in S_i$ and each node $n \in X$, the maximal time $f_i(n, X) \in \mathbb{N}$ associated with $n$ and $X$. From $S_{i+1}$ and $f_i$, we inductively compute $f_{i+1}$ in the following way: for all $X \in S_i$ with successor $Y \in S_{i+1}$ for outcomes $(r_p)_{p\in P}$, we denote $f_{i+1}(Y, n, X) = \max_{p\in P(n)} f_i(X, n(X, p)) + \gamma^+(n(X, p), r_p)$. If there are several choices of $(r_p)_{p\in P}$ leading to the same $Y$, we take $r_p$ with the maximal $f_i(X, n(X, p)) + \gamma^+(n(X, p), r_p)$. We then define $f_{i+1}(Y, n) = \max_{X\in S_i} f_{i+1}(Y, n, X)$. Again, the initialization is trivial, with $f_0(\{n_0\}, n_0) = 0$. The maximal execution time of $\mathcal{N}$ is $f(\{n_f\}, n_f)$. □

We can bound the complexity precisely by $O(d(\mathcal{N}) \cdot C(\mathcal{N}) \cdot ||R||^{k^*})$, with:

- $d(\mathcal{N}) \leq |\mathcal{N}|$ the depth of $n_f$, that is the number of layers of $\mathcal{N}$, and $||R||$ is the maximum number of outcomes of a node,
- $C(\mathcal{N}) = \max_i |S_i| \leq 2^k$, which we will call the *number of contexts of* $\mathcal{N}$, and which is often much smaller than $2^k$.
- $k^* = \max_{X\in\bigcup_i S_i} |X| \leq k$. We say that $\mathcal{N}$ is $k^*$*-thread bounded*, meaning that there cannot be more that $k^*$ nodes in the same context $X$ of any layer. Usually, $k^*$ is strictly smaller than $k = \max_i |N_i|$, as $N_i = \bigcup_{X\in S_i} X$.

Consider again the Brexit example Figure 1. We have $(k + 1) = 7$, while we have the depth $d(\mathcal{N}) = 6$, the negotiation is $k^* = 3$-thread bounded ($k^*$ is bounded by the number of processes), $||R|| = 2$, and the number of contexts is at most $C(\mathcal{N}) = 4$ (EU chooses to enforce backstop or not, and Pa chooses to go to court or not).

### 7.2 Minimal Execution Time

As with sound negotiations, computing minimal time is much harder than computing the maximal time for $k$-layered negotiations:

**Theorem 7.** *Let $k \geq 6$. The Min $\leq T$ problem is NP-Complete for k-layered acyclic negotiations, even if the negotiation is sound and very weakly non-deterministic.*

*Proof.* One can guess in polynomial time a final run of size $\leq |\mathcal{N}|$. If the execution time of this final run is smaller than $T$ then we have found a final run witnessing $mintime(\mathcal{N}) \leq T$. Hence the problem is in NP.

Let us now show that the problem is NP-hard. We proceed by reduction from the **Knapsack** decision problem. Let us consider a set of items $U = \{u_1, \dots u_n\}$ of respective values $v_1, \dots v_n$ and weight $w_1, \dots, w_n$ and a knapsack of maximal capacity $W$. The knapsack problem asks, given a value $V$ whether there exists a subset of items $U' \subseteq U$ such that $\sum_{u_i\in U'} v_i \geq V$ and such that $\sum_{u_i\in U'} w_i \leq W$.

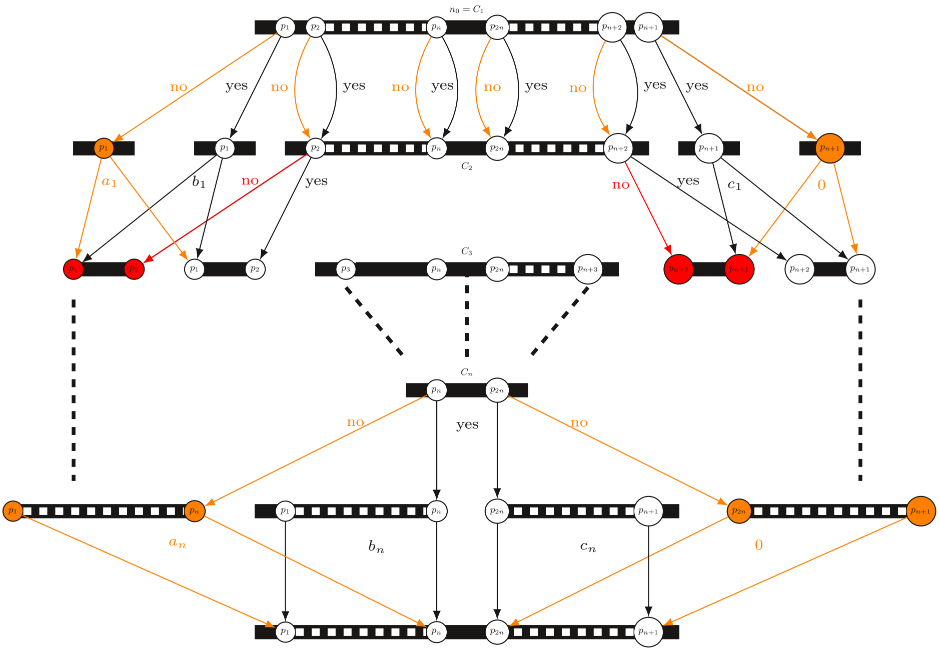

**Fig. 5.** The negotiation encoding Knapsack

We build a negotiation with $2n$ processes $P = \{p_1, \dots p_{2n}\}$, as shown in Fig. 5. Intuitively, $p_i, i \leq n$ will serve to encode the value of selected items as timing, while $p_i, i > n$ will serve to encode the weight of selected items as timing.

Concerning timing constraints for outcomes we do the following: Outcomes 0, yes and no are associated with $[0, 0]$. Outcome $c_i$ is associated with $[w_i, w_i]$, the weight of $u_i$. Last, outcome $b_i$ is associated with a more complex function, such that $\sum_i b_i \leq W$ iff $\sum_i v_i \geq V$. For that, we set $[\frac{(v_{max}-v_i)W}{n \cdot v_{max}-V}, \frac{v_{max}W}{n \cdot v_{max}-v_i}]$ for outcome $b_i$, where $v_{max}$ is the largest value of an item, and $V$ is the total value we want to reach at least. Also, we set $[\frac{(v_{max})W}{n \cdot v_{max}-V}, \frac{v_{max}W}{n \cdot v_{max}-v_i}]$ for outcome $a_i$. We set $T = W$, the maximal weight of the knapsack.

Now, consider a final run $\rho$ in $\mathcal{N}$. The only choices in $\rho$ are outcomes *yes* or *no* from $C_1, \dots, C_n$. Let $I$ be the set of indices such that *yes* is the outcome from all $C_i$ in this path. We obtain $\delta(\rho) = \max(\sum_{i \notin I} a_i + \sum_{i \in I} b_i, \sum_{i \in I} c_i)$. We have $\delta(\rho) \leq T = W$ iff $\sum_{i \in I} w_i \leq W$, that is the sum of the weights is lower than $W$, and $\sum_{i \notin I} \frac{(v_{max})W}{n \cdot v_{max}-V} + \sum_{i \in I} \frac{(v_{max}-v_i)W}{n \cdot v_{max}-V} \leq W$. That is, $n \cdot v_{max} - \sum_{i \in I} v_i \leq n \cdot v_{max} - V$, i.e. $\sum_{i \in I} v_i \geq V$. Hence, there exists a path $\rho$ with $\delta(\rho) \leq T = W$ iff there exists a set of items of weight less than $W$ and of value more than $V$. $\square$

It is well known that Knapsack is weakly NP-hard, that is, it is NP-hard only when weights/values are given in binary. This means that Thm. 7 shows that minimum execution time $\leq T$ is NP-hard only when $T$ is given in binary. We can actually show that for $k$-layered negotiations, the $mintime(\mathcal{N}) \leq T$ problem can be decided in PTIME if $T$ is given in unary (i.e. if $T$ is not too large):

**Theorem 8.** *Let $k \in \mathbb{N}$. Given a $k$-layered negotiation $\mathcal{N}$ and $T$ written in unary, one can decide in PTIME whether the minimum execution time of $\mathcal{N}$ is $\leq T$. The worst-case time complexity is $O(|\mathcal{N}| \cdot |P| \cdot (T \cdot |\mathcal{N}|)^k)$.*

*Proof.* We will remember for each layer $i$ a set $\mathcal{T}_i$ of functions $\tau$ from nodes $N_i$ of layer $i$ to a value in $\{1, \ldots, T, \perp\}$. Basically, we have $\tau \in \mathcal{T}_i$ if there exists a path $\rho$ reaching $X = \{n \in N_i \mid \tau(n) \neq \perp\}$, and this path reaches node $n \in X$ after $\tau(n)$ time units. As for $S_i$, for all $p$, we should have a unique node $n(\tau, p)$ such that $p \in n(\tau, p)$ and $\tau(n(\tau, p)) \neq \perp$. Again, it is easy to initialize $\mathcal{T}_0 = \{\tau_0\}$, with $\tau_0(n_0) = 0$, and $\tau_0(n) = \perp$ for all $n \neq n_0$.

Inductively, we build $\mathcal{T}_{i+1}$ in the following way: $\tau_{i+1} \in \mathcal{T}_{i+1}$ iff there exists a $\tau_i \in \mathcal{T}_i$ and $r_p \in R_{n(\tau_i,p)}$ for all $p \in P$ such that for all $n$ with $\tau_{i+1}(n) \neq \perp$, we have $\tau_{i+1}(n) = \max_p \tau_i^-(n(\tau_i, p)) + \gamma(n(\tau_i, p), r_p)$.

We have that the minimum execution time for $\mathcal{N}$ is $\min_{\tau \in \mathcal{T}_n} \tau(n_\tau)$, for $n$ the depth of $n_f$. There are at most $T^k$ functions $\tau$ in any $\mathcal{T}_i$, and there are at most $|\mathcal{N}|$ layers to consider, giving the complexity. □

As with Thm. 6, we can more accurately state the complexity as $O(d(\mathcal{N}) \cdot C(\mathcal{N}) \cdot ||R||^{k^*} \cdot T^{k^*-1})$. The $k^* - 1$ is because we only need to remember minimal functions $\tau \in \mathcal{T}_i$: if $\tau'(n) \geq \tau(n)$ for all $n$, then we do not need to keep $\tau'$ in $\mathcal{T}_i$. In particular, for the knapsack encoding in the proof of Thm. 7, we have $k^* = 3$, $||R|| = 2$ and $C(\mathcal{N}) = 4$. Notice that if $k$ is part of the input, then the problem is strongly NP-hard, even if $T$ is given in unary, as e.g. encoding bin packing with $\ell$ bins result to a $2\ell + 1$-layered negotiations.

## 8 Conclusion

In this paper, we considered timed negotiations. We believe that time is of the essence in negotiations, as examplified by the Brexit negotiation. It is thus important to be able to compute in a tractable way the minimal and maximal execution time of negotiations. We showed that we can compute in PTIME the maximal execution time for acyclic negotiations that are either sound or $k$-layered, for $k$ fixed. We showed that we cannot compute in PTIME the maximal execution time for negotiations that are not sound nor $k$-layered, even if they are deterministic and acyclic (unless NP=PTIME). We also showed that surprisingly, computing the minimal execution time is much harder, with strong NP-hardness results in most of the classes of negotiations, contradicting a claim in [10]. We came up with a new reasonable class of negotiations, namely $k$-layered negotiations, which enjoys a pseudo PTIME algorithm to compute the minimal execution time. That is, the algorithm is PTIME when the timing constants are given in unary. We showed that this restriction is necessary, as the problem becomes NP-hard for constants given in binary, even when the negotiation is sound and very weakly non-deterministic. The problem to know whether the minimal execution time can be computed in PTIME for deterministic and sound negotiation remains open.
